# Investigating the factor structure of a translated recovery-orientation instrument in inpatient treatment for substance use disorder

**DOI:** 10.1186/s13011-021-00363-0

**Published:** 2021-03-19

**Authors:** Dagny Adriaenssen Johannessen, Amy Østertun Geirdal, Trond Nordfjærn

**Affiliations:** 1 Blue Cross East, Oslo, Norway; 2 Department of Social Work, Child Welfare and Social Policy, OsloMet – Oslo Metropolitan University, Oslo, Norway; 3 Department of Psychology, Norwegian University of Science and Technology (NTNU), Trondheim, Norway

**Keywords:** Confirmatory factor analysis, Recovery-orientation, Recovery self-assessment, Substance use disorder, Translate/back-translate

## Abstract

**Background:**

Recovery has been outlined as a process of change through which involvement and empowerment enables individuals to reach their goals and aspirations. Recovery self-assessment (RSA) is an instrument that has been acknowledged as an applicable measure of recovery-orientation in services for people with mental health problems or substance use disorder (SUD). This study aimed to translate RSA from US English to Norwegian and to investigate the factor structure of the translated version (RSA-N).

**Methods:**

A translate/back-translate procedure was used. Confirmatory factor analysis (CFA) was applied to investigate the factor structure of RSA-N in a sample of clinicians (*n* = 407) working in inpatient SUD treatment facilities.

**Results:**

The results suggested that the hypothesised five-factor structure originally obtained by the developers showed an inadequate fit with the current data sample. RSA-N was modified and restructured by removing twelve misfitting items and combining factors with high covariance using data from one subsample. The alternative three-factor structure yielded an acceptable fit for the data from a second subsample. Acceptable alpha coefficients, suggesting good internal consistency, supported the adequacy of the three-factor structure.

**Conclusions:**

Results from the present study are in line with previous findings, which have failed to replicate the hypothesised five-factor structure without modifications. Knowledge about the degree to which SUD services are recovery-oriented may contribute to SUD services’ pursuit of establishing an inpatient treatment environment that fosters change and development of inpatients. The present study’s findings imply RSA-N’s potential as an instrument to assess recovery-orientation in inpatient SUD treatment.

**Supplementary Information:**

The online version contains supplementary material available at 10.1186/s13011-021-00363-0.

## Background

In the course of time, recovery-orientation has been established as part of public services for people with substance use disorder (SUD) or mental health problems [1–4]. Recovery communities are occupied with how people who experience negative consequences of substance use or poor mental health are perceived and approached. Recovery has been outlined as a process of change where involvement and empowerment facilitate the necessary autonomy, self-perception and belonging to reach individually defined goals and to live a life that the individual finds meaningful [5–7]. Recovery may thus be regarded as a goal or a process, as well as a measure to establish an environment that fosters change and development [5, 6]. Recovery as a goal implies leading a meaningful life as defined by the individual and as a process, recovery involves changes in life domains that are affected by the negative consequences of substance use or mental health problems [8]. Recovery as a measure refers to a framework that promotes the process and the goal of recovery, often implemented by services in their pursuit of becoming recovery-oriented services [7].

Several instruments have been developed to measure the degree to which SUD and mental health services are recovery-oriented [3, 9]. One is Recovery self-assessment (RSA: [10]), a validated self-report instrument with satisfactory internal consistency, which has been an applicable measure of recovery-orientation and acknowledged as such [3]. RSA was developed in the United States (US) to measure recovery-orientation in community, outpatient or inpatient services. Separate versions of RSA are available for different target groups, comprising users, family members, service providers and managers. RSA can be used in one or several of these target groups to assess recovery-orientation in services or for research purposes.

RSA for users has previously been translated from US English to Chinese [11], Swedish [12], Malaysian [13] and Hong Kong Chinese [14], in which all have been tested for validity among people attending community mental health services. More recently, RSA for users has been translated to Brazilian Portuguese and culturally adapted to community mental health services in Brazil but has not yet been psychometrically tested [15].

The providers’ version of RSA has previously been translated from US English to German [16] and tested for internal consistency among the staff of a psychiatric inpatient hospital [17]. Validity studies have revealed diverging psychometric properties of the providers’ version of RSA in psychiatric inpatient hospital settings. Salyers, Tsai and Stultz [18] found that RSA had good internal consistency and stable test-retest reliability. In contrast, Thege, Ham and Ball [19] failed to confirm the hypothesised factor structure in their data sample.

The providers’ version of RSA has been used to explore associations between mental health clinicians’ perception of the degree to which services are recovery-oriented, on one hand, and different factors, such as job satisfaction, stigmatising attitudes, therapeutic alliance with service users, and recovery outcome among users, on the other hand [20–24]. However, RSA has hardly been used to study recovery-orientation in specialised SUD services. During the development of RSA, staff from both mental health and SUD services participated, but the results were aggregated [10]. To the best knowledge of this paper’s authors, no studies have used RSA to assess clinicians’ perception of recovery-orientation specifically in SUD services or have investigated its validity in a population of clinicians working in specialised inpatient SUD treatment facilities.

Specialised treatment for SUD may be considered a planned turning point where basic needs are met and development and change are initiated [25]. Inpatient SUD treatment has been recognised as particularly important for individuals with SUD who have multiple psychosocial challenges and struggle in managing their everyday life [26–28]. Recovery-orientation has been established as an expedient way to organise and provide services for individuals who undergo extensive changes [29–31]. RSA captures several of the enabling factors in the vast change process that is often involved in recovering from SUD, including social support, belonging, motivation, involvement and predictability in terms of basic needs and rate of change [32–38].

People with SUD are more often targeted with stigmatising attitudes compared to people with mental health problems [39, 40]. Stigmatising attitudes also appear among clinicians working in the treatment of mental health problems or SUD [41]. A recovery-oriented framework includes measures to counter common negative beliefs about people with SUD or mental health problems. These measures involve addressing stigmatising descriptions of people with SUD or informing the general public about SUD and its recovery potential. Such measures may contribute to reduced stigma and discrimination, both among clinicians [42, 43] and the general public [39, 44]; A wider assessment of the recovery-orientation in SUD services is therefore significant.

Few instruments are suitable for assessing the degree of recovery-orientation in SUD services, as perceived by clinicians, and with qualities similar to those of RSA. RSA may be a useful tool in exploring recovery-orientation in inpatient SUD treatment.

This study had two aims: 1) to formally translate the providers’ version of RSA from US English to Norwegian and 2) to investigate the factor structure of the translated version among clinicians working in inpatient SUD treatment facilities.

## Materials and methods

### Recovery Self-Assessment (RSA)

RSA includes 36 items, which are rated on a 5-point Likert scale (from 1 = “strongly disagree” to 5 = “strongly agree”), with two additional options (N/A = “not applicable” and D/K = “don’t know”). The items are divided into five subscales that provide information about the services’ ability to promote service users’ recovery. The subscales are hypothesised to measure the extent to which service users’ *Life goals*, *Involvement*, and *Choice* are promoted by the service staff. The instrument also covers the degree to which the services offer a *Diversity of treatment options* and are *Individually-tailored services*. The instrument provides individual scores for each of the five subscales, as well as a total score. Scores on the individual subscales yield information about potential areas of improvement to establish a recovery-oriented environment in the treatment facility or service. High scores suggest that the services are recovery-oriented, while low scores indicate the opposite [10].

### Translation procedure

RSA was translated according to a translate/back-translate procedure and guidelines for translation [45] (see Table 1).

**Table 1:** Translation procedure according to Wild and colleagues’ [45] guidelines

***Procedure***	Description
*Preparations*	The literature was searched for Norwegian translations and the originator of RSA was informed about the research project
*Forward translation*	RSA was translated from US English to Norwegian by two native Norwegian speakers with clinical and research experience in mental health and SUD
*Revise*	The forward-translation was adapted to the Norwegian inpatient SUD treatment setting in terms of language, culture and organisation of health services
*Back translation*	The forward-translation was back-translated from Norwegian to US English by a professional translator with no knowledge of the original instrument
*Review*	The back-translation was reviewed and compared to the original instrument
*Harmonization*	The authors discussed differences in the original- and back translated versions in terms of conceptual correspondence and adjusted the forward-translated version
*External assessment*	Clinicians completed the instrument and commented on wording, concepts, understandability and relevance of the included items
*Adjustment*	The translated version was adjusted according to feedback from the clinicians
*External assessment and proofreading*	NGO-representatives commented on language, concepts, understandability, relevance of the instrument and proofread the instrument
*Adjustment and finalisation*	The adjusted translation was finalised according to feedback from the NGO-representatives

In the preparation phase, the five RSA subscales were thoroughly reviewed to evaluate if they captured the factors that have previously been outlined as enablers in the vast change process involved in recovering from SUD. The RSA developers do not require stakeholders to obtain permission to use the instrument (i.e., no copyright) [3, 46]. However, the originator was informed about the research project and the plans for translation before the procedure started.

RSA was forward-translated from US English to Norwegian by two of the authors (DAJ and AØG), who have clinical and research experience in mental health and SUD and are native Norwegian speakers. The forward-translation was adapted to the Norwegian inpatient SUD treatment setting in terms of language, culture and organisation of health services. The translated version was then back-translated by a professional translator with no knowledge of the original instrument.

The back-translation was reviewed and compared with the original instrument. The authors discussed the differences between the original and the back-translated versions in terms of conceptual correspondence and adjusted the forward-translated version accordingly.

The adjusted forward-translated version was first sent to clinicians (*n* = 6) working in a specialised outpatient SUD treatment programme. They were asked to complete the instrument and comment on the wording, concepts, understandability and relevance of the included items. Their responses were reviewed, and the translated version was adjusted according to their feedback.

The adjusted version was then sent to representatives (*n* = 4) of *non-governmental organisations* (NGO) in the SUD field for further assessment. They were asked to comment on the language, concepts, understandability and relevance of the instrument. They were also instructed to proofread the instrument. The adjusted translation was then modified according to their feedback, and the instrument was established as *Recovery Self-Assessment – Norwegian* (RSA-N). The translation procedure took place from January to July 2020.

### Setting and participants

#### Setting

Regarding this study’s second aim, the factor structure was investigated in Norway, where specialized outpatient and inpatient SUD treatments are organised under four regional health enterprises (Northern Norway Regional Health Authority, Central Norway Regional Health Authority, Western Norway Regional Health Authority, and Southern and Eastern Norway Regional Health Authority). These health enterprises award private organisations with contracts to provide specialised SUD treatment. The contractual agreement ensures that the private providers adhere to formal requirements. The expenses for inpatient SUD treatment are covered with public funds, and people who undergo outpatient treatment pay a small deductible.

In line with those of other western countries, Norway’s specialised inpatient SUD treatment is interdisciplinary and consists of psychological, social and medical interventions and measures. Administered in the treatment facility where the patients reside, inpatient SUD treatment normally comprises individual, environmental and group therapies [47–49]. To adhere to the ideal of interdisciplinarity in inpatient SUD treatment facilities, psychologists, social workers, nurses, medical doctors and psychiatrists are normally employed.

Inpatient SUD treatment facilities in Norway adhere to various therapeutic orientations. Some provide treatment that originates from the psychodynamic tradition, such as mentalisation-based therapy. Others provide treatment from the recovery tradition. Among these are Hierarchical Therapeutic Communities (CTC) and inpatient twelve-step programmes. Several of the facilities are *treatment collectives*. These have a democratic structure, like Democratic Therapeutic Communities (DTC), but originated partly through inspiration from pedagogical collectives for adolescents with behavioural difficulties in Sweden and the Soviet Union. Lastly, other inpatient SUD treatment facilities adhere to cognitive behavioural therapy or describe their therapeutic orientation as *eclectic*.

#### Participants

The study protocol was independently reviewed and approved by the Norwegian Centre for Research Data (NSD; reference number 883511). The participants received written information and gave their consent by answering the first item in the questionnaire: “I give my consent to participate in the study and to my answers being stored in Sensitive Data Services (TSD) and used for the purpose of research.”

Fifty-four eligible treatment facilities were invited to participate in the study. Among these, 50 facilities responded to the invitation, and a contact person from each facility provided clinical staff members with a link to a self-report questionnaire via e-mail. Additionally, potential respondents received one or two reminders to answer the questionnaire. Ideally, an equal number of participants from all facilities should be included. However, to attain a sufficient number of participants, all clinical staff members at each facility were invited. The participating facilities employed from 10 to 50 clinicians in total (mean = 15, median = 16). More participants therefore contributed from larger facilities than from smaller facilities. In total, 426 of the 933 (response rate = 46%) invited clinicians completed the questionnaire; 96% (*n* = 407) of these respondents reported that they worked directly (i.e., clinically) with the inpatients in the treatment facility. The respondents who did not work clinically (*n* = 19) were excluded from the study. The participants comprised clinicians working in long-term (≥ 6 months) inpatient SUD treatment facilities in Norway. The data were collected from August to October 2020.

### Investigating the factor structure

#### Measures

The questionnaire consisted of RSA-N and six items that collected demographic information, including age, gender, number of years spent working in the SUD field, number of years spent working in an SUD treatment facility, and job title. One of the items from the original RSA concerned accessibility by collecting information about the place where the services were provided. During inpatient treatment, the inpatient’s reside in the treatment facility; thus, the item was removed, as suggested by Campbell-Orde, Chamberlin, Carpenter and Stephen Leff [46]. The RSA-N therefore consisted of 35 items, which were formulated as statements about the treatment programme. The questionnaire was completed by assessing the 35 RSA-N items on a 5-point Likert scale (for the scoring options, see above), as well as the six demographic items, which took approximately 15 min to complete.

The total RSA-N score was obtained by computing the mean of all 35 items (ranging from 1 to 5). The individual subscale scores were obtained by computing the mean of the items included in each subscale (ranging from 1 to 5), as suggested by the originators [10].

#### Analytical strategy

Confirmatory factor analysis (CFA) is normally applied when the structure of a measurement instrument, such as RSA, has been established through assessing which items are pooled together in a latent factor (i.e., a subscale) [50]. CFA was therefore conducted to investigate whether the hypothesised five-factor structure of the original RSA, as described by O’Connell, Tondora, Croog, Evans and Davidson [10], coincided with the factor structure of RSA-N in the current data sample. Due to poor fit indices, the sample was randomly divided into two equally sized subsamples, which provided an opportunity to modify RSA-N with a sample that differed from the one used to test the validity of the modified RSA-N (alternative model).

Principal component analysis (PCA) and modification indices in CFA may be used to remove items and respecify the factor structure of an instrument [50]. Therefore, the data from subsample 1 were used to identify cross-loading items, items with weak loadings (i.e., misfitting items) and covariance between hypothesised factors using PCA and modification indices in CFA. The data from subsample 2 were used to test the validity of the alternative model.

The chi-square test and approximate fit indices designate how well the data fit the hypothesised factor structure (measurement model) by using information about common and unique variances of the indicators (items and latent factors) obtained with the observed data. The fit indices estimated the model fit by placing the observed data on a range from a poor model (baseline model), with no information about variance, to a good model (saturated model), with all the information about variance.

The chi-square (χ^2^) test, root mean square error of approximation (RMSEA) with 90% confidence interval (90% CI) and comparative fit index (CFI) were used to evaluate the fit of the hypothesised factor structure in the current data sample. In the chi-square test, small differences between the data and the hypothesised model are preferred, and *p*-values above 0.05 may indicate a good fit. The chi-square test is sensitive to both small and large sample sizes [51]. The two approximate fit indices, RMSEA and CFI, were therefore emphasised in evaluating the adequacy of the hypothesised five-factor structure for the current data. A low RMSEA is desirable, and values above 0.08 and an upper confidence interval value above 0.1 may indicate a poor fit [52]. A high CFI is desirable, and the fit may be acceptable with values close to 0.90 [53]. Internal consistency was considered using Cronbach’s alpha coefficients (α), which estimate the adequacy of pooling designated items together to measure a latent construct or a subscale. Alpha coefficients with values of 0.7 and above indicate acceptable internal consistency for the data [51].

Descriptive statistics were used to examine the respondents’ personal and professional characteristics, as well as patterns of system-missing values (additional response options N/A and D/K). Mean imputation, as described by Christophersen [54] (the item mean value plus the subscale mean value divided by two), was used on items with 90% or more valid responses [55]. All analyses were conducted both with and without mean imputation, providing the same results in terms of factor structure and misfitting items.

Pattern analyses of system-missing values were performed using IBM SPSS Statistics Version 27, while jamovi version 1.2.27 was used for all other analyses [56].

## Results

### Sample characteristics

The respondents’ personal and professional characteristics are presented in Table 2. The participants’ mean age was 44.7 years, and 68% were women. The mean time spent working in the SUD field was 9.96 years, and the mean time spent working in an inpatient SUD treatment facility was 7.7 years. Among the participants, 35% were medical staff members, 16% served as social workers, 11% worked as psychologists or therapists, and 38% were registered as *other staff*, including peer specialists, environmental therapists, financial counsellors and job counsellors. Four items (4, 27, 12 and 15) had more than 10% system-missing values, with valid responses ranging from *n* = 319 to *n* = 378.

**Table 2:** Respondents’ personal and professional characteristics

	n (%)	Total *n* = 407 mean (SD)
**Gender**		
Women	275 (68)	
Men	132 (32)	
**Age**		44.7 (10.6)
20–30	44 (12)	
31–40	99 (26)	
41–50	121 (31)	
51–60	97 (25)	
> 60	23 (6)	
**Job title**		
Medical staff	145 (35)	
Social worker	64 (16)	
Psychologist and therapist	44 (11)	
Other staff	154 (38)	
**Years spent working in the SUD field**		9.96 (7.38)
< 5	120 (30)	
5–10	131 (32)	
11–15	69 (17)	
16–20	50 (12)	
> 20	37 (9)	
**Years spent working with SUD treatment**		7.70 (6.45)
< 5	164 (40)	
5–10	139 (34)	
11–15	52 (13)	
16–20	33 (8)	
> 20	19 (5)	

CFA was conducted to investigate how well the five-factor structure, originally obtained by O’Connell, Tondora, Croog, Evans and Davidson [10], would fit the current data. The RMSEA (90% CI) values (0.052 (0.048–0.056)) suggested an adequate fit, while the CFI (0.083) indicated a poor fit (chi-square test: χ^2^ (df) = 1155.982 (550), *p* < 0.01). Additionally, the factor covariance between *Life Goals* and *Choice* was high (0.95), as was the factor covariance between the two hypothesised factors: *Diversity of treatment options* and *Individually-tailored services* (0.94). High covariation between factors indicates related or overlapping factors, which is fairly common in multidimensional psychometric instruments. However, high values normally imply that the model has poor discriminant validity and should be respecified [50].

Due to these results, the study group was randomly divided into two equal subsamples: subsample 1 (*n* = 203) and subsample 2 (*n* = 204). RSA-N was modified and respecified using PCA and modification indices in CFA with the data from subsample 1. The modification resulted in an alternative model, which was tested with CFA using the data from subsample 2.

### Modifying RSA-N

As some correlations between the components were expected, oblique rotation with Promax was applied during the PCA. Bartlett’s test of Sphericity (χ^2^ (df) = 1496.080 (595), *p* < 0.01) and Kaiser-Meyer-Olkin (KMO) of Sampling Adequacy (MSA = 0.78) implied that the data were suitable for PCA [57].

The items that did not load with values over 0.3 on a component in the PCA were removed, as were the cross-loading items with values over 0.4 [50]. One item (29, see Table 3) did not load over 0.3, and none of the items cross-loaded.

**Table 3:** Removed items

Item	***Statement***
1.	*Helping the patients build connections with their neighbourhoods and communities is one of the primary activities in which staff at this agency are involved*
3.	*The patients have access to all their treatment records*
4.	*This agency provides education to community employers about employing people with mental illness and/or addictions*
6.	*The patients can choose and change, if desired, the therapist, psychiatrist, or other service provider with whom they work*
12.	*This agency provides structured educational activities to the community about mental illness and addictions*
13.	*Agency staff do not use threats, bribes, or other forms of coercion to influence the patient’s behaviour or choices*
16.	*Staff are knowledgeable about special interest groups and activities in the community*
19.	*This agency provides a variety of treatment options (*i.e.*, individual, group, peer support, holistic healing, alternative treatments, medical) from which the patients may choose*
20.	*The achievement of goals by patients and staff are formally acknowledged and celebrated by the agency*
26.	*Agency staff are diverse in terms of culture, ethnicity, lifestyle, and interests*
29.	*Staff routinely assist patients in the pursuit of educational and/or employment goals*
32.	*This agency provides formal opportunities for patients, family members, service providers, and administrators to learn about recovery*

Ten items (1, 4, 6, 12, 13, 16, 19, 20, 26 and 32; see Table 3) with weak loading (< 0.4) on the designated factor or high loadings on several factors were identified and removed one by one, using modification indices in CFA [50].

### Respecifying the factor structure

The original factor structure was respecified by investigating common and unique variances between the items and the latent factors in CFA. The covariance between two pairs of the hypothesised factors – *Life goals* and *Choice*, and *Diversity of treatment options* and *Individually-tailored service*s – remained high.

*Life goals* consist of items addressing staff members’ role in helping inpatients outline and achieve their individually defined goals, such as “Staff actively assist patients with the development of career and life goals that go beyond symptom management and stabilization.” *Choice* includes items that gather information about the extent to which staff members use measures to influence inpatients’ choices connected to defining their individual goals, such as “Staff at this agency listen to and follow the choices and preferences of the patients.”

*Diversity of treatment options* contains items that collect information about the degree to which various treatment options are offered and whether the treatment programme is varied in terms of inpatients’ individual needs, such as “Groups, meetings and other activities can be scheduled in the evenings or on weekends so as not to conflict with other recovery-oriented activities, such as employment or school.” *Individually-tailored services* comprise items that gather information about the extent to which the treatment programme is customised to meet inpatients’ individual needs, such as “This agency offers specific services and programmes for individuals with different cultures, life experiences, interests and needs.”

The two factors – *Life goals* and *Choice* – were combined and established as *Goals and choice*. Likewise, *Individually-tailored services* and *Diversity of treatment options* were merged and established as *Individually tailored and varied* (for the alternative three-factor solution, see Additional file 1). Model fit indices for the alternative three-factor model showed improved results. RMSEA (90% CI) (0.055 (0.043–0.064)) and CFI (0.87) indicated an acceptable fit (chi-square test: χ^2^ (df) = 394.5516 (249), *p* < 0.01).

### Testing the alternative model

**Fig. 1:**
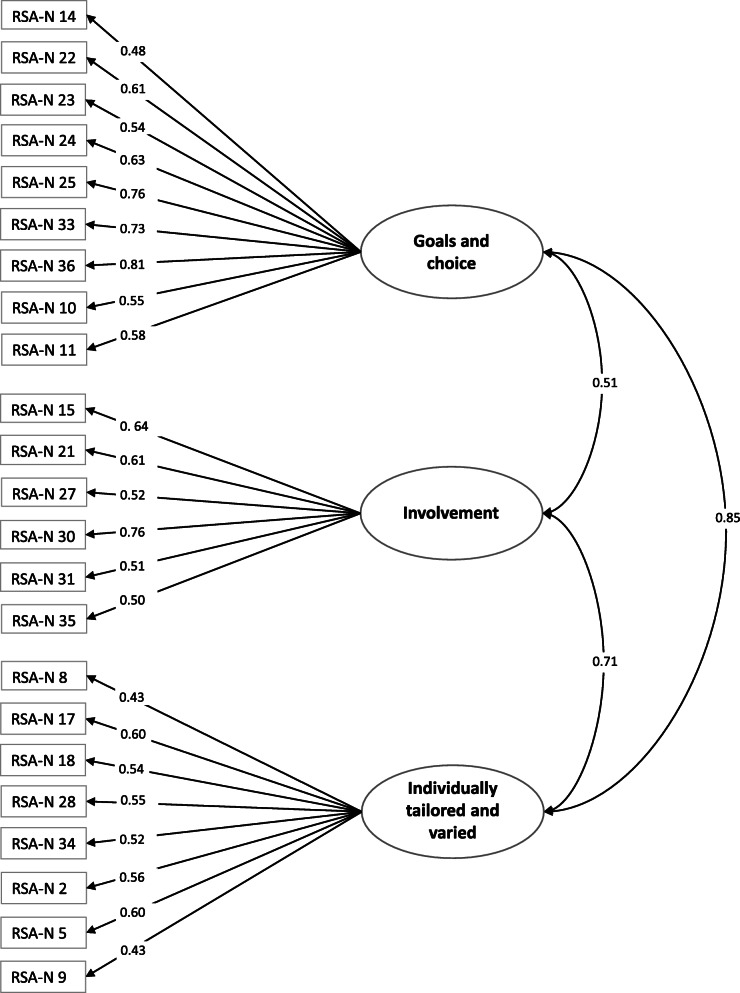
Alternative three-factor model

The alternative three-factor structure was tested using CFA with data from subsample 2. The results are presented in Fig. 1. One item (3, see Table 3) was removed due to weak loading (< 0.4).

Model fit indices indicated that the alternative factor structure was applicable, with RMSEA (90% CI) (0.059 (0.049–0.069)) and CFI (0.89) both indicating an acceptable fit (chi-square test: χ^2^ (df) = 388.1141 (227), *p* < 0.01).

The factor loadings (standardised) ranged from 0.43 to 0.81 and were significant (*p* < 0.05). The covariance (standardised) between the factors ranged from 0.51 to 0.85 and were significant (*p* < 0.05). Reliability analyses indicate good internal consistency for the three factors – *Goals and choice* (α = 0.82), *Involvement* (α = 0.74) and *Individually tailored and varied* (α = 0.75) – and for the overall RSA-N instrument (α = 0.88).

## Discussion

The main results of the present study are that the five-factor structure originally obtained by O’Connell, Tondora, Croog, Evans and Davidson [10] has an inadequate fit with the current data from the SUD sector. Twelve misfitting items were removed during the modification, and factors with high covariance were combined. An alternative three-factor model is proposed for RSA-N, comprising the subscales *Goals and choice*, *Involvement* and *Individually tailored and varied*. Approximate fit indices for the alternative model were found to be acceptable, as was the internal consistency according to alpha coefficients.

This present study’s main findings coincide with prior validity studies with translated versions of RSA, which have found acceptable internal consistency for the subscales included in the users’ version of RSA [11–13, 17] and fair to good internal consistency for the subscales included in the providers’ version [17, 18].

In line with the present study’s results, previous studies of the factor structure have failed to replicate the hypothesised five-factor structure without modifications. Based on the CFA results, Tan and Fernandez [13] concluded that the five-factor structure could be replicated by allowing the error terms of eight items to covary. Tan and Fernandez [13] study is the only one that has successfully replicated the original five-factor structure using a translated version of RSA. Furthermore, based on their results from a Rasch analysis, the authors suggested that the *Life Goals* subscale should be split into two, which they recommended for further use in the Chinese version of RSA [11]. Based on exploratory factor analysis with parallel analysis, Ye, Pan, Wong and Bola [14] suggested that three factors could be extracted, and Lodge, Kuhn, Earley and Manser [58] reported that a one-factor solution provided the best fit for the data. Although Ye, Pan, Wong and Bola [14] found a three-factor solution with their data sample, due to their research aims, they decided to use a one-factor solution and therefore did not provide supplementary information about the three-factor solution. Lastly, Thege, Ham and Ball [19] tested the psychometric properties of the original RSA with CFA. Based on unacceptable fit indices and high covariance between several of the latent factors, they concluded that the original five-factor structure had an inadequate fit in a sample of staff members working with inpatient treatment for severe mental illness.

None of these prior studies have tested the hypothesised factor structure in a sample of clinicians working with inpatient SUD treatment. Furthermore, we found no previous studies that explored the adequacy of using RSA to assess recovery-orientation in SUD services in general or inpatient SUD treatment in particular since the RSA was developed.

Several factors may explain the issue with replicating the original five-factor structure shown in this and previous validation studies of RSA. First, there are variations in how people with mental health problems are approached compared to people with SUD. The most common notion of recovery applied in the European SUD field today is derived from movements such as the twelve-step tradition and sobriety movements, where eliminating the addictive substance (i.e., the symptom) represents a core value [59–61]. In the mental health field, however, the prevailing notion of recovery developed as part of a civil rights movement. The movement emerged during the 1960s and consisted of people with mental health problems who advocated their right and opportunity to take part in society on equal terms to other citizens, regardless of their symptoms [2]. The main differences in treatment approaches between these recovery orientations may be exemplified through two frameworks: DTCs, which are more common in treating mental health problems, and CTCs, which are generally applied when treating SUD [60]. In DTCs, psychodynamic principles are applied to enhance the patients’ understanding of their current reaction patterns, considering their attachment style and their past experiences. CTCs, on the other hand, are more influenced by reward theory. Rewards and consequences are employed to change behaviours that are perceived to inhibit patients’ recovery [60]. The variations in these approaches may be useful to illustrate differences in conventional perceptions about how the two conditions develop and progress. Such variations in conventional attitudes could have influenced the participants’ reports on the RSA in this and previous studies.

Second, the way SUD and mental health problems are perceived and treated differs between cultures and countries. For instance, there are cultural variations in patterns of use, perceived harm, prevalence and acceptance of substances [62]. Additionally, differences in knowledge about causes, symptoms or the way the condition progresses may cause variations in how mental health problems are perceived in different cultures [63, 64]. Krendl and Pescosolido [64] illustrate this in their discussion. Based on their findings, the authors hypothesise that mental health conditions that are identified as treatable may generate a perception that low social function among people with mental health problems is their own choice. Cultural variations like these might have contributed to the issue with replicating the original five-factor structure shown in this and previous translations of the RSA.

Lastly, previous research has shown that there are major differences in how the notion of recovery is understood among clinicians and between services [65–67]. The clinicians in this study were not asked to outline their perceptions of recovery. However, variations in how recovery is understood may influence the implementation of recovery-orientation and which recovery measures are emphasised above others at the treatment facility.

Previous studies that have used RSA to examine recovery-orientation have been conducted in services both for people with mental health problems and for SUD. These studies have shown that the therapeutic orientation may influence the culture at the treatment facility and the clinicians’ perceptions in various ways. Some studies showed that a higher degree of recovery-orientation at the treatment facility was associated with better work-related satisfaction [68], more positive attitudes towards patients [69] and less stigmatising attitudes among clinicians [42]. Other studies have shown positive associations between recovery-orientation and the treatment team of clinicians in terms of positive attitudes towards patients, better team climate and higher trust between team members [70, 71]. We could not find any previous studies that solely used the RSA in specialised SUD services.

Considering this, the present study contributes to existing research by investigating RSA-N’s potential as an instrument for assessing recovery-orientation exclusively in specialised SUD treatment. This contribution may induce a wider assessment of the degree to which SUD services and inpatient SUD treatment are recovery-oriented.

Some limitations of the current study should be considered. First, the data were obtained using a self-report questionnaire. Despite the advantages of using a self-report questionnaire, the risk of social desirability bias is present. The questionnaire was completed anonymously by the participants, which reduced the risk of social desirability bias. Second, the response rate was acceptable but moderate (46%). Also, larger facilities contributed with more responses compared to smaller facilities. However, participants from 50 out of the 54 eligible inpatient SUD treatment facilities in Norway responded to the questionnaire, which indicates that the data comprised a broad representation of the target population. Additionally, the sample size provided the opportunity to modify RSA-N and test the validity of the alternative model in two separate subsamples of the original sample. Lastly, the CFI value was close to but did not exceed 0.90 when testing the alternative model with the data from subsample 2. The CFI could be improved by letting the error terms of several items to covary. However, this was not done due to the limitations of such an approach [50]. Additionally, the RMSEA values obtained for the alternative three-factor model suggested a good fit. Similarly, the alpha coefficients indicated satisfactory internal consistency for the three-factor RSA-N solution.

## Conclusions

Since RSA was introduced in 2005, the instrument has been extensively used to assess the degree to which mental health services are recovery-oriented. However, RSA has hardly been used to assess recovery-orientation in SUD services and inpatient SUD treatment. In the current study, an alternative three-factor structure of the Norwegian translation – RSA-N – has obtained an acceptable fit and good internal consistency in a sample of clinicians working in inpatient SUD treatment facilities. The results are primarily supported by findings from earlier investigations of the instrument’s psychometric properties [11, 13, 14, 19, 58] and correspond with the results of previous examinations of its internal consistency [11–13, 18]. People with SUD are often stigmatised and discriminated in the broader society. Recovery-oriented measures have been known to reduce stigma and discrimination; therefore, it is significant that SUD services evaluate to what extent their practice is recovery-oriented. Knowledge about the degree to which SUD services are recovery-oriented, as well as knowledge about factors facilitating recovery-orientation in SUD services, may be important contributions in stigma prevention and in establishing an SUD treatment environment that fosters change and development of inpatients. The present study’s findings imply RSA-N’s potential as an instrument to assess recovery-orientation in inpatient SUD treatment.

## Supplementary Information

**Additional file 1.** Recovery Self-Assessment – Norwegian (RSA-N).

## Data Availability

The datasets generated and/or analysed during the current study are not publicly available due to ethical restrictions but are available from the corresponding author on reasonable request.

## Abbreviations

CTC: Hierarchical Therapeutic Community
CFA: Confirmatory factor analysis
CFI: Comparative fit index
DTC: Democratic Therapeutic Community
NGO: Non-governmental organisations
RMSEA: Root mean square error of approximation
PCA: Principal component analysis
RSA: Recovery Self-Assessment
RSA-N: Recovery Self-Assessment – Norwegian
SUD: Substance use disorder
