# Correction to: Investigating the factor structure of a translated recovery-orientation instrument in inpatient treatment for substance use disorder

**DOI:** 10.1186/s13011-021-00411-9

**Published:** 2021-09-22

**Authors:** Dagny Adriaenssen Johannessen, Amy Østertun Geirdal, Trond Nordfjærn

**Affiliations:** 1 Blue Cross East, Oslo, Norway; 2 Department of Social Work, Child Welfare and Social Policy, OsloMet – Oslo Metropolitan University, Oslo, Norway; 3 Department of Psychology, Norwegian University of Science and Technology (NTNU), Trondheim, Norway; 4 St. Olavs Hospital, Clinic of Substance Use and Addiction Medicine, Trondheim, Norway

**Correction to: Subst Abuse Treat Prev Policy 16, 24 (2021)**

**https://doi.org/10.1186/s13011-021-00363-0**

In the original publication [1], the following affiliation was missing for Trond Nordfjærn

- 4 St. Olavs Hospital, Clinic of Substance Use and Addiction Medicine, Trondheim, Norway

The original article has been updated.
