# Supplementary material for: Investigating the factor structure of a translated recovery-orientation instrument in inpatient treatment for substance use disorder
Source: Subst Abuse Treat Prev Policy. 2021 Mar 19;16:24. doi: 10.1186/s13011-021-00363-0 (PMC7980679; doi:10.1186/s13011-021-00363-0)
Supplement: Supplementary file 1 — Additional file 1. Recovery Self-Assessment – Norwegian (RSA-N). [file 13011_2021_363_MOESM1_ESM.docx]

**Appendix A:** Alternative three-factor solution (RSA-N)

| **Goals and choice** | |
| --- | --- |
| 10. | Staff at this agency listen to and follow the choices and preferences of the patients. |
| 11. | Progress made towards goals (as defined by the patient) is monitored on a regular basis. |
| 14. | Staff and patients are encouraged to take risks and try new things. |
| 22. | Staff use a language of recovery (i.e. hope, high expectations, respect) in everyday conversations. |
| 23. | Staff play a primary role in helping patients become involved in non-mental health/addiction related. |
| 24. | Procedures are in place to facilitate referrals to other programs and services if the agency cannot meet a patients’ needs. |
| 25. | Staff actively assist patients with the development of career and life goals that go beyond symptom management and stabilisation. |
| 33. | The role of agency staff is to assist patients with fulfilling their individually-defined goals and aspirations. |
| 36. | Agency staff believe that people can recover and make their own treatment and life choices. |
| **Involvement** | |
| 15. | The patients are involved with facilitating staff trainings and education programs at this agency. |
| 21. | The patients are routinely involved in the evaluation of the agency’s programs, services, and service providers. |
| 27. | The patients are regular members of agency advisory boards and management meetings. |
| 30. | The patients work alongside agency staff on the development and provision of new programs and services. |
| 31. | Agency staff actively help patients become involved with activities that give back to their communities (i.e., volunteering, community services). |
| 35. | The development of patient’s leisure interests and hobbies is a primary focus of services. |
| **Individually tailored and varied** | |
| 2. | This agency offers specific services and programs for individuals with different cultures, life experiences, interests, and needs. |
| 5. | Every effort is made to involve significant others (spouses, friends, family members) and other natural supports (i.e., clergy, neighbours, landlords) in the planning of the patient’s treatment, if so desired. |
| 8. | The patients are given the opportunity to discuss their sexual and spiritual needs and interests. |
| 9. | All staff at this agency regularly attend trainings on cultural competency. |
| 17. | Groups, meetings, and other activities can be scheduled in the evenings or on weekends so as not to conflict with other recovery-oriented activities such as employment or school. |
| 18. | This agency actively attempts to link patients with other persons in recovery who can serve as role models or mentors by making referrals to self-help, peer support, or consumer advocacy groups or programs. |
| 28. | At this agency, patients who are doing well get as much attention as those who are having difficulties. |
| 34. | Criteria for exiting or completing the treatment program are clearly defined and discussed with patients upon entry to the agency. |
